# Methodological quality of teaching communication skills to undergraduate medical students: a mapping review

**DOI:** 10.1186/s12909-018-1265-4

**Published:** 2018-06-27

**Authors:** Rob Sanson-Fisher, Breanne Hobden, Amy Waller, Natalie Dodd, Lucy Boyd

**Affiliations:** 1 Health Behaviour Research Collaborative, School of Medicine and Public Health, Faculty of Health and Medicine, University of Newcastle, Callaghan, NSW Australia; 2 Priority Research Centre for Health Behaviour, Faculty of Health and Medicine, The University of Newcastle, Callaghan, New South Wales 2308 Australia; 3 Hunter Medical Research Institute, New Lambton, New South Wales Australia

**Keywords:** Communication skills, Physician/patient relationship, Quantitative research methods, Continuing medical education

## Abstract

**Background:**

Patient-clinician communication training is a core component of the undergraduate medical program. As with all areas of medicine, the best available evidence for teaching these skills should be incorporated into training programs. Examining the volume, type and design-quality of publications in this field can help to determine whether research is following a natural scientific progression to inform interactional skills training. This study aimed to review: (i) whether the proportion of publications examining teaching interactional skills to undergraduate medical students by study type, across three time-periods (2007–2008, 2011–2012, 2015–2016), changed over time (i.e. measurement, descriptive or interventions studies); and (ii) the proportion of intervention studies meeting Cochrane Effective Practice and Organisation of Care (EPOC) research design criteria.

**Methods:**

Medline, PubMed, PsycInfo and the Cochrane Database were searched for studies published in English from 2007 to 2016. Title and abstract reviews were performed for the included years. Articles were examined against the inclusion/exclusion criteria and those included were coded into descriptive, measurement or intervention categories.

**Results:**

A total of 243 relevant publications were identified. Fifty-two were published from 2007 to 2008, 75 from 2011 to 2012 and 116 from 2015 to 2016**.** Most identified studies were descriptive (63%), followed by measurement studies (22%) and intervention studies (15%). The proportion of descriptive studies increased significantly over time. However, the proportion of intervention studies did not change and the proportion of measures studies significantly decreased. Of the 37 intervention studies identified within the three time-periods, only 16 (43%) met EPOC study design criteria.

**Conclusions:**

The largest proportion of identified studies were descriptive, however, descriptive research is not sufficient to ensure communication skills training can effectively improve interactions between clinicians and patients. A more rigorous approach to research in this area is needed to inform education strategies.

**Electronic supplementary material:**

The online version of this article (10.1186/s12909-018-1265-4) contains supplementary material, which is available to authorized users.

## Background

### Description of patient-clinician communication

The Institute of Medicine’s landmark ‘Crossing the Quality Chasm’ report, describes free and open sharing of knowledge between patients and clinicians as one of the tenets of optimal patient-centred care [1]. The extent to which this can be achieved largely depends on the quality of patient-clinician communication. While difficult to define, it is generally accepted that patient-clinician communication incorporates three broad domains. These domains include: information gathering which can aid diagnosis; information transfer which involves providing information to the patient regarding treatment options or adherence to health regimes; and general interactional skills, covering areas such as empathy, professional etiquette and control of available consultation time [2]. Additional elements of patient-clinician interaction include building the relationship [3], harmonised goals and transparency as well as full disclosure [4].

### The importance of patient-clinician communication

Effective patient-clinician communication can enhance patient satisfaction [5, 6], improve health outcomes [7], and improve adherence to treatment plans [8]. Poor communication can limit patient understanding of their illness or treatment [9], lead to poorer patient outcomes, or to complaints against services and clinicians [9, 10]. Poor communication and failing to understand the patient’s perspective are consistently reported as responsible for more complaints than any other domain, including misdiagnosis [10–12]. Common law standards in many countries now indicate a need for clinicians to provide information to patients about their condition and choices regarding medical care in a way that the patient can understand [13–15]. The value of good communication skills has been highlighted by clinicians [16], health care recipients [17] and medical education accreditation bodies [18–20]. Recognising the importance of having junior doctors that can demonstrate an acceptable standard of communication skills [21] has renewed interest in communication skills training in undergraduate medical education.

### There is a need to demonstrate the effectiveness of undergraduate medical communication skills training

A Lancet publication in 1980 highlighted the potential benefit of teaching communication skills to undergraduate medical students [22]. With the emergence of communication skills training as a core component of the undergraduate medical curriculum, there is a need to be able to demonstrate that the wide range of communication skills perceived as necessary for appropriate clinical practice can be feasibly taught, acquired and transferred to a clinical setting. This includes evidence that this educational process leads to changes in the behaviour of health care clinicians. Some previous research has indicated that communication skills can be taught and learned in both simulated and actual clinic environments [23, 24]. Nevertheless, ongoing rates of consumer dissatisfaction with clinician communication and non-adherence to medical advice suggest that clinicians are not consistently acquiring or maintaining these skills [2]. There is a strong impetus for those working in the field to determine where gaps in current practice are occurring, and potential strategies that may help establish strong evidence for the feasibility and effectiveness of communication skills training.

### The volume, type and methodological quality of publication output allows for assessment of research effort in the field

Examining the research involving undergraduate medical training in communication skills provides insight to whether the research has followed a natural scientific progression to inform existing training programs. For instance, it may be expected that the development of robust assessment measures to test skills and outcomes of future research is the first step to conducting research in this field [25, 26]. Such measures include the Liverpool Undergraduate Communication Assessment Scale which can test communication skills during Objective Structured Clinical Examinations (OSCEs) [27]. Once robust assessment measures are available to quantify communication skills in undergraduate medical students, descriptive research (cross-sectional, prospective and retrospective study designs) [28] can be used to identify barriers and enablers to teaching under-graduate communication skills. Next, intervention research should test strategies that address descriptive findings to ascertain the methods that are most effective at teaching communication skills [25, 26]. These interventions should be performed in such a way that ensure rigour, scientific integrity and methodological quality. Therefore, examining the type, volume and quality of the published research allows for critical assessment of the progression of the field.

### Aims

To systematically review: (i) whether the proportion of publications examining teaching interactional skills to undergraduate medical students by study type, across three time-periods (2007–2008, 2011–2012, 2015–2016), changed over time (i.e. measurement, descriptive or interventions studies); and (ii) the proportion of intervention studies meeting Cochrane Effective Practice and Organisation of Care (EPOC) research design criteria.

## Methods

### Data sources

Medline, EMBASE, PsycINFO and the Cochrane Database were searched for studies published in English from 2007 to 2016.

### Search strategy

The following search terms were used: ‘communication skills’, ‘interpersonal communication’, ‘communication skills training’, ‘physician-patient relations’, ‘medical students’, ‘undergraduate’, ‘medical education’, ‘teaching’, ‘curriculum’, ‘competency-based education’, ‘educational measurement’, ‘clinical competence’. The comprehensive search strategy can be found in Additional file 1.

### Inclusion/exclusion criteria

Inclusion criteria: Publications that: i) examined interactional skills for undergraduate medical students. Undergraduate medical students were defined as college or University students undertaking a degree in medicine. Interactional skills related to the following domains: information gathering (e.g. taking a history, forming diagnosis); information transfer (e.g. cessation, preparing patients for potentially threatening procedures, breaking bad news); or overall interaction skills (e.g. opening and closing consultations as well as interpersonal skills); ii) included an outcome measure examining behavioural change (intervention studies); iii) were published in English in a peer-reviewed journal; iv) published in 2007–2008, 2011–2012 or 2015–2016. This time period was selected as it was thought to be sufficient for examining the trend of recent publications in the field.

Exclusion criteria: Studies were excluded if they: (i) were focussed on inter-professional interaction skills; (ii) were not focussed on the undergraduate medical degree (i.e. involved only residents or postgraduate specialities such as psychiatry); or (iii) were qualitative research, case studies or non-databased studies. Criterion three was established as these types of studies are generally not considered to contribute to high levels of evidence [29].

### Description of study types

Articles meeting the inclusion criteria were coded according to their publication types. These were data-based publications reporting new data, including measurement, descriptive or intervention studies.

### Classification of articles

Title and abstract review was performed against the inclusion criteria and publications were coded according to study type. One author and a research assistant individually coded 10 papers at a time until an agreement rate of 80% was reached. This was performed for the first 40 papers in 2016. The research assistant then continued with the remainder of the coding and the author (BH) assessed a random subsample of at least 10% for each year to examine ongoing agreement. Any discrepancies were discussed between the research assistant and one author (BH). When agreement dropped below 80%, another random subsample were assessed. The kappa statistic demonstrated an overall agreement rate for inclusion of 83.47% across all years (κ = .6179; *P* < .001).

### EPOC criteria for intervention studies

Two authors (BH and LB) reviewed the intervention studies against the minimal EPOC design criteria [30]. This includes: randomised controlled trials (RCTs), non-randomised controlled trials (N-RCTs), controlled before-after study and interrupted time series. Any discrepancies were resolved between authors.

### Data analysis

A linear regression analysis of the volume of publications versus time was conducted. Cochran-Armitage Trend Tests were used to determine whether there was a change over time of the proportion of publications examining teaching interactional skills overall and classified by study type, i.e. measurement, descriptive or intervention. A *p*-value of < 0.05 was used to indicate significance. All analyses were conducted in SAS software for Windows version 9.4.

## Results

**Fig. 1:**
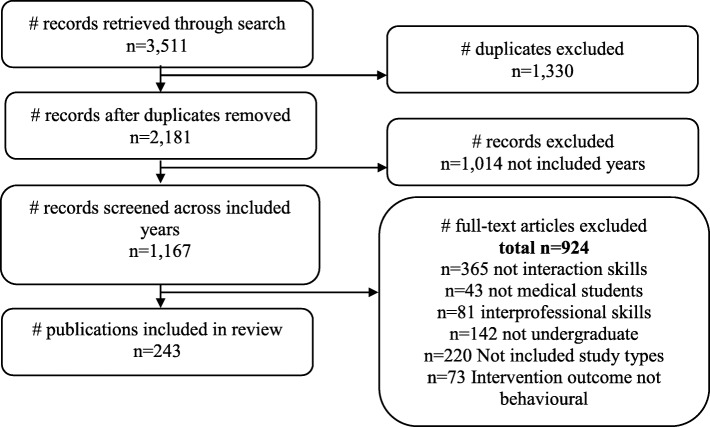
Study flow chart

A total of 3511 citations were retrieved using the search strategy and 2181 were assessed after duplicates were removed. Across the included years there were 1167 citations and of these 243 relevant studies were included (see Fig. 1). Of the 243 identified relevant publications examining interaction skills, 52 were published from 2007 to 2008, 75 from 2011 to 2012 and 116 from 2015 to 2016. The change in the total volume of publications increased by approximately 32 per year, however, this was not statistically significant (*p* = 0.10).

### Proportion of publications by study-type and changes over time (Fig. 2)

When examining the publication designs, the majority of studies were descriptive (62.6%, *n* = 152). There were a total of 54 measures papers (22.2%) and 37 intervention papers (15.2%) across the three time-points. There was a statistically significant decrease over time in the proportion of publications that were classified as measurement papers (Cochran-Armitage Trend Test Z = − 3.0491, *p* = 0.0022). The proportion of descriptive research increased over time and this was statistically significant (Cochran-Armitage Trend Test Z = 2.1084, *p* = 0.0359). There was no significant increase for the proportion of intervention studies (Cochran-Armitage Trend Test Z = 0.688, *p* = 0.5122).

**Fig. 2:**
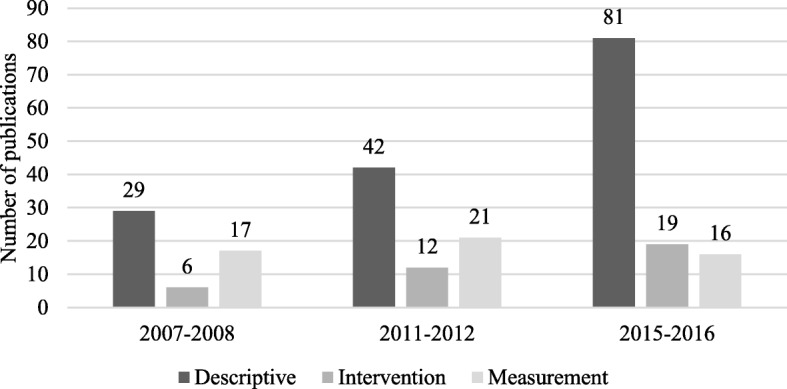
Number of publications by study-type (*n* = 243)

### Proportion of intervention studies meeting EPOC study design criteria

There were 37 publications reporting interventions. Of these 16 met EPOC study design criteria including 4 RCTs and 12 N-RCTs. The remaining 21 interventions used non-EPOC study-design criteria. Across the three time periods, 3/6 (50%) interventions met EPOC design criteria from 2007 to 2008, 5/12 (42%) from 2011 to 2012 and 8/19 (42%) from 2015 to 2016.

## Discussion

This paper examined three time-periods during the past 10 years to determine where published research efforts have been focused in the field of educating undergraduate medical students in communication skills. Of the 243 publications identified, almost two thirds were descriptive studies, 22% were measurement studies, while only 15% of publications were intervention studies. The research effort directed toward empirical work in interactional skills training across the three selected time periods did not demonstrate a clear scientific progression. If this were the case, there would be an increasing proportion of intervention studies and a decreasing proportion of descriptive research. This was not demonstrated in the current review.

### Publications by study-type and changes over time

A natural scientific progression of research within almost any field would firstly require the development of robust research measures [26]. This would be followed by descriptive data to test measures and build empirical knowledge. Finally, intervention research should be conducted to allow for testing of initiatives to improve the delivery of evidence-based practice [26].

Publications relating to measures demonstrated a significant decrease over the three time-points. While there was a decline in the volume of measurement studies, this does not appear to be clearly related to the development of psychometrically robust measures, as a wide range of outcome measures were used in the intervention studies. In contrast, descriptive research consistently represented the majority of the research during each time period, with the proportion of descriptive research increasing significantly over time. Descriptive studies can provide important information about current practice of communication skills [6, 21], including data on how well students are performing across the various components of interactional skills training. Such data can then be used to identify components where poorer outcomes are occurring and to refine training programs so that greater attention is paid to these components [25]. A key limitation of this descriptive research is that it only provides correlational data. For the field to progress, it needs to move beyond collecting descriptive data. Instead, intervention research is needed to provide requisite causation data to establish the effectiveness of programs in improving interactional skills.

The small number of intervention studies was consistent across the three time points. This finding highlights a potential lack of progression in the literature. It is important to note that a majority of the intervention studies in this field were excluded from this review as they examined interaction skills without using a behavioural outcome measure. Most of these intervention studies instead relied on outcomes such as satisfaction and knowledge as evidence of efficacy. This is a significant limitation of the literature in this field, as only through examining behavioural outcomes can we ensure that programs are achieving a meaningful change in students’ clinical communication skills. Even when a behavioural outcome was included, the measures used to assess these outcomes in the included studies varied widely [31, 32]. Without standardised measures, it is not possible to combine and compare data across intervention trials in a meaningful way.

### Proportion of intervention studies meeting EPOC study design criteria

Less than half of the intervention studies met the EPOC study design criteria [30]. Well-controlled intervention studies are critical if we are to establish that communication skills training can change the interaction between medical students and patients and improve outcomes. High quality randomised controlled trials are needed to provide currently missing Level I evidence for interactional skills training in areas such as breaking bad news, empathy, information gathering and provision, and preventive health guidance.

### Future research

Communication skills training for medical undergraduates must be built on a foundation of strong scientific evidence. As a first step, measures of clinical behaviour for intervention research should be considered. It is important to establish measures that are able to determine meaningful change in observed behaviours, as well as demonstrating high inter-rater reliability. Further, improvements in the volume and quality of intervention research are paramount to provide causal knowledge and level one evidence to inform training. There are several areas in which new intervention research should focus attention. Understanding the characteristics/qualities of teachers that are the most successful in skills training may help to enhance transferability of skills to students. For instance, students may be more inclined to respond to the importance of interaction skills if they are taught such skills from a practicing clinician. Examining the cost-effectiveness of interactional skills training could help to inform resource allocation and ensure that teaching these skills has an economic benefit. Understanding when interactional skills should be administered throughout the undergraduate program could assist with course design. It may be reasonably argued that a general focus on interaction skills is introduced in the beginning of the course, with more explicit skills provided in the later years [33]. However, there is a need for evidence supporting that this is the most effective way to impart these skills if this is how they are to be incorporated in the curricula [34, 35]. A focus on information transfer, as well as information gathering is also required. While interaction skills are commonly associated with gathering history and disease information [2–4], there is a need to ensure that clinicians are able to adequately and clearly relay information back to their patients. Lastly, there is a need to examine whether skills generalise across time, i.e. from undergraduate, to resident to practicing clinician; and across different clinical settings, such as surgery as well as psychiatry [36]. Researchers should ensure robust methods are used and intervention trials are reported in sufficient detail to allow replication in a real-world setting [37]. Providing standardisation in course administration can help to ensure that all students are receiving adequate training, which is evidence-based and shown to improve real-world application of interactional skills.

### Limitations

This review should be viewed in light of several limitations. The restriction of examining literature within the three time periods were selected to provide an overview of the trend of this literature, however, the authors acknowledge that these times periods are relatively arbitrary and are not representative of all the data in this topic area. This review also did not include grey literature, dissertations or policy documents. While this is a potential limitation, it is likely that high quality research would be published in peer-reviewed journals and therefore captured in our search strategy. Furthermore, while this review does not attempt to extract and summarise the findings of the intervention research which was returned, it serves to act as a cue to researchers in the field that there is broad pattern of a lack of rigorous intervention research. It highlights a clear need to increase output and methodological quality in this area as the returned literature indicates that the small number of interventions and lack of standardised measurement would not enable meaningful comparison of the data collected to date. This is a commonly reported short-coming of publications in the Cochrane Database of Systematic Reviews.

## Conclusion

Communication skills have been reported to be important within the medical system by researchers, consumers and clinicians. If communication skills continue to be an integral component of undergraduate medical programs, curricula must be informed by best-evidence. Most of the identified studies in this review were descriptive studies, however, descriptive research is not enough to demonstrate which training programs are most effective in improving students’ skills. Furthermore, less than half of the identified intervention studies met rigorous intervention design criteria. Future researchers are tasked with ensuring a more rigorous approach to research in this area is applied, particularly for intervention studies.

## Additional file

Additional file 1: Search Strategy. The database search strategy used to identify relevant articles is included in this document. (DOC 32 kb)

## Abbreviations

EPOC: Cochrane effective practice and organisation of care
N-RCTs: Non-randomised controlled trials
RCT: Randomised controlled trials
