# Supplementary material for: Methodological quality of teaching communication skills to undergraduate medical students: a mapping review
Source: BMC Med Educ. 2018 Jun 27;18:151. doi: 10.1186/s12909-018-1265-4 (PMC6020352; doi:10.1186/s12909-018-1265-4)
Supplement: Supplementary file 1 — Search Strategy. The database search strategy used to identify relevant articles is included in this document. (DOC 32 kb) [file 12909_2018_1265_MOESM1_ESM.doc]

**Appendix 1. Search Strategy.**

Database: Medline <1946 - present>

Search Strategy:

--------------------------------------------------------------------------------

1 Communication/ or Physician-patient relations/ (131460)

2 ((communicat* or interaction*) adj2 (skill* or assess*)).tw. (14678)

3 1 or 2 (141442)

4 Medical students/ (27837)

5 "medical student*".tw. (33123)

6 ((undergraduate* or student*) adj2 (medical or medicine)).tw. (37193)

7 4 or 5 or 6 (48285)

8 exp Education, Medical/ (149412)

9 ((communicat* or interaction*) adj4 (train* or educat* or curricul* or framework or teach* or program* or workshop* or instruct*)).tw. (17386)

10 exp Teaching/ (78246)

11 curriculum/ or competency-based education/ (70200)

12 educational measurement/ or clinical competence/ (105477)

13 8 or 9 or 10 or 11 or 12 (313222)

14 3 and 7 and 13 (3029)

15 limit 14 to (english language and yr="2007 -Current") (1545)

16 limit 15 to (case reports or editorial or letter or news) (83)

17 15 not 16 (1462)

Database: Embase <1947 to present>

Search Strategy:

--------------------------------------------------------------------------------

1 doctor patient relation/ (110880)

2 *interpersonal communication/ or communication skill/ (53201)

3 ((communicat* or interaction*) adj2 (skill* or assess*)).tw. (18967)

4 1 or 2 or 3 (168116)

5 medical student/ (56628)

6 "medical student*".tw. (41151)

7 ((undergraduate* or student*) adj2 (medical or medicine)).tw. (46201)

8 5 or 6 or 7 (70907)

9 teaching/ (84875)

10 medical education/ (204049)

11 clinical education/ (11965)

12 education/ (373208)

13 curriculum/ (76773)

14 clinical competence/ (51688)

15 ((communicat* or interaction*) adj2 (train* or educat* or curricul* or framework or teach* or program* or workshop* or instruct*)).tw. (11110)

16 9 or 10 or 11 or 12 or 13 or 14 or 15 (636830)

17 4 and 8 and 16 (3652)

18 limit 17 to (english language and yr="2007 -Current") (2099)

19 limit 18 to (books or "book review" or chapter or conference abstract or conference paper or "conference review" or editorial or letter or note) (631)

20 18 not 19 (1468)

Database: PsycINFO <1806 to June Week 3 2017>

Search Strategy:

--------------------------------------------------------------------------------

1 interpersonal communication/ (14029)

2 communication skills/ or communication skills training/ (8758)

3 therapeutic processes/ (22786)

4 (communicat* adj2 (skill* or assess*)).tw. (11113)

5 (interact* adj2 (skill* or assess*)).tw. (3113)

6 1 or 2 or 3 or 4 or 5 (52801)

7 training/ (12763)

8 exp medical education/ (21757)

9 exp teaching/ (107058)

10 ((communicat* or interaction*) adj4 (train* or educat* or curricul* or framework or teach* or program* or workshop* or intervention* or instruct*)).tw. (28985)

11 7 or 8 or 9 or 10 (160026)

12 medical students/ (12119)

13 "medical student*".tw. (13596)

14 ((undergraduate* or student*) adj2 (medical or medicine)).tw. (14808)

15 12 or 13 or 14 (17185)

16 6 and 11 and 15 (879)

17 limit 16 to (english language and yr="2007 -Current") (463)

18 limit 17 to (chapter or editorial or letter) (33)

19 17 not 18 (430)

20 limit 19 to "0200 book" (2)

21 19 not 20 (428)

Database: Cochrane

Search Name:

Date Run: 26/06/17 04:15:09.8

Description:

ID Search Hits

#1 MeSH descriptor: [Communication] this term only 1855

#2 MeSH descriptor: [Physician-Patient Relations] this term only 1288

#3 communicat* near/2 (skill* or assess*):ti,ab,kw (Word variations have been searched) 1065

#4 interaction* near/2 (skill* or assess*):ti,ab,kw (Word variations have been searched) 386

#5 #1 or #2 or #3 or #4 3854

#6 MeSH descriptor: [Students, Medical] this term only 757

#7 "medical student*":ti,ab,kw (Word variations have been searched) 2261

#8 undergraduate* near/2 (medical or medicine):ti,ab,kw (Word variations have been searched) 748

#9 student* near/2 (medical or medicine):ti,ab,kw (Word variations have been searched) 2525

#10 #6 or #7 or #8 or #9 2651

#11 MeSH descriptor: [Education, Medical] explode all trees 2810

#12 communicat* near/4 (train* or educat* or curricul* or framework or teach* or program* or workshop* or instruct*):ti,ab,kw (Word variations have been searched) 1477

#13 interaction* near/4 (train* or educat* or curricul* or framework or teach* or program* or workshop* or instruct*):ti,ab,kw (Word variations have been searched) 620

#14 MeSH descriptor: [Teaching] explode all trees 3589

#15 MeSH descriptor: [Curriculum] this term only 1201

#16 MeSH descriptor: [Competency-Based Education] this term only 80

#17 MeSH descriptor: [Educational Measurement] this term only 1413

#18 MeSH descriptor: [Clinical Competence] this term only 2677

#19 #11 or #12 or #13 or #14 or #15 or #16 or #17 or #18 9267

#20 #5 and #10 and #19 153
